# Supplementary figures and images for: An Insight of Betula platyphylla SWEET Gene Family through Genome-Wide Identification, Expression Profiling and Function Analysis of BpSWEET1c under Cold Stress
Source: Int J Mol Sci. 2023 Sep 4;24(17):13626. doi: 10.3390/ijms241713626 (PMC10488219; doi:10.3390/ijms241713626)

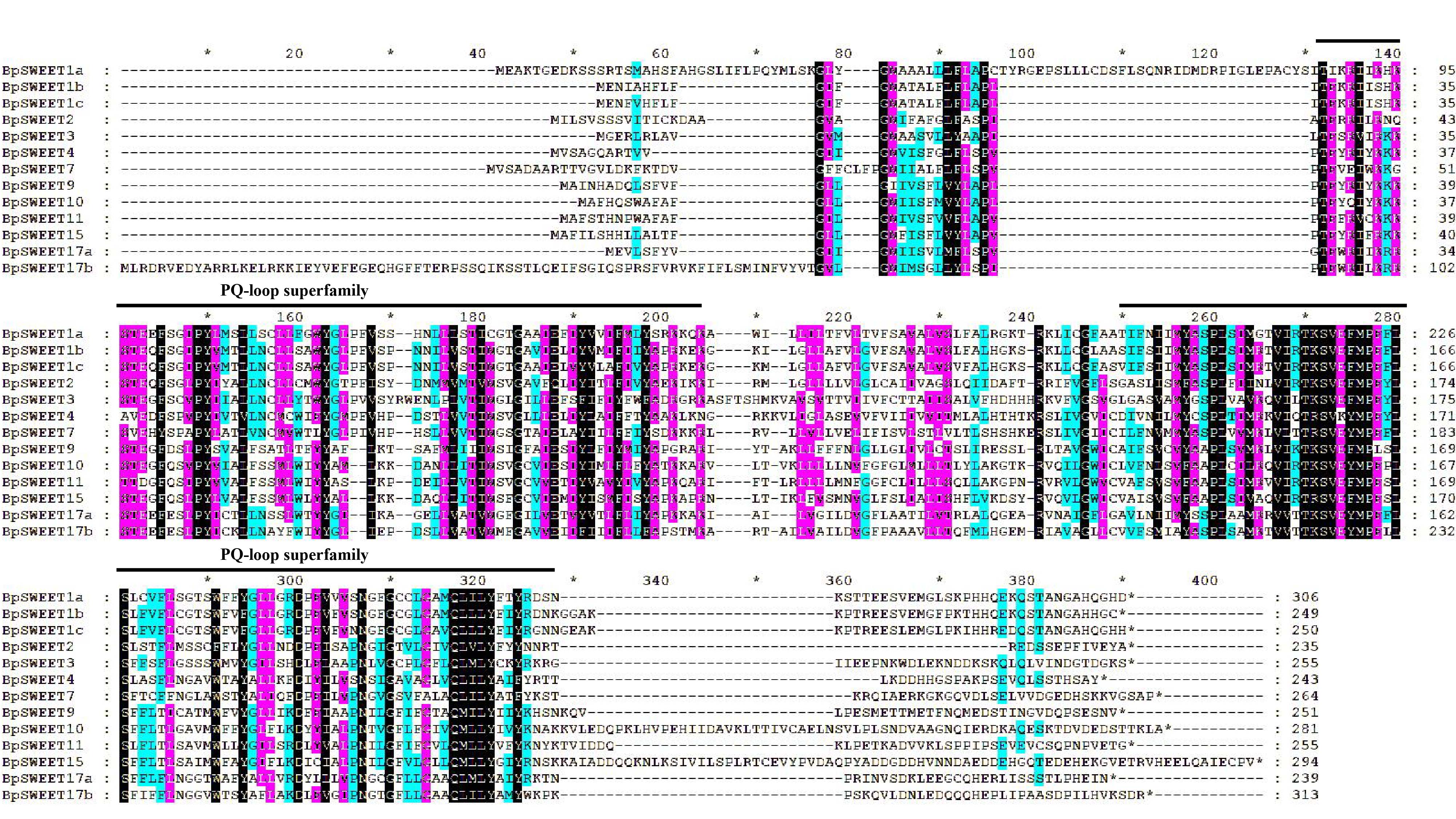

Supplement: Supplementary file 1 [file ijms-24-13626-s001.zip › FigureS1.jpg]

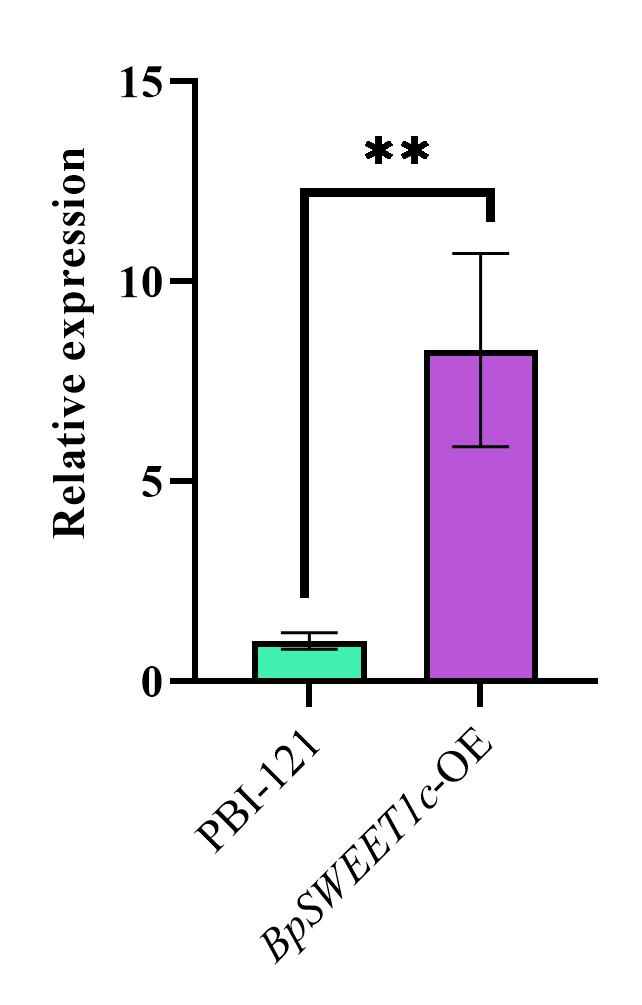

Supplement: Supplementary file 1 [file ijms-24-13626-s001.zip › FigureS2.jpg]

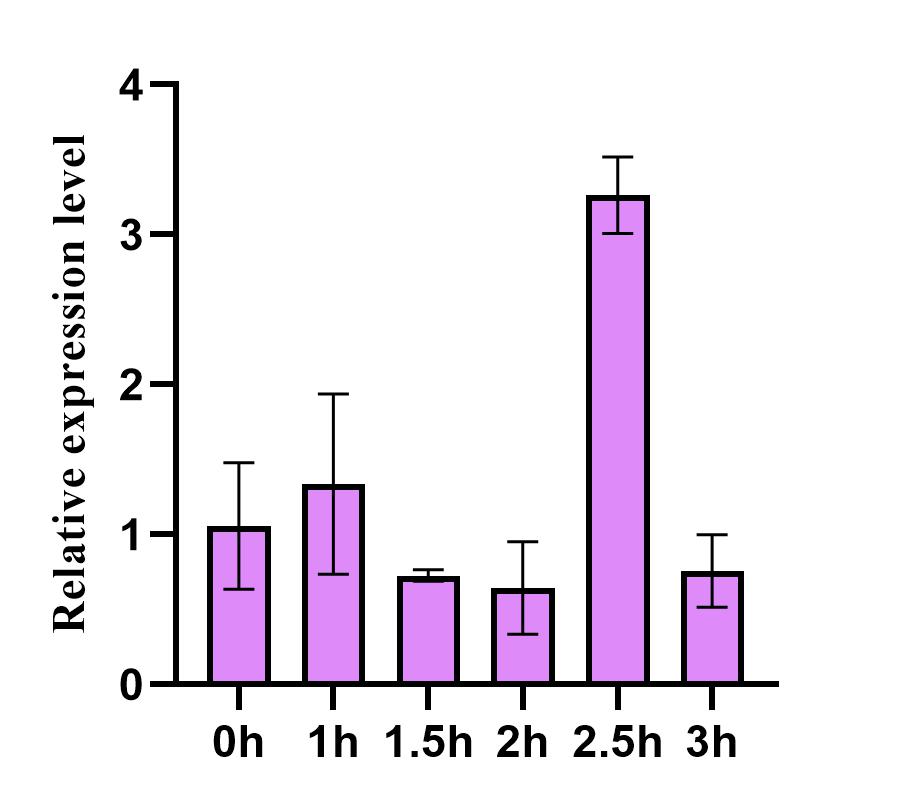

Supplement: Supplementary file 1 [file ijms-24-13626-s001.zip › FigureS3.jpg]
